# PIN‐PMN‐PT Surface Planar Waveguides Realized via He^+^ Implantation

**DOI:** 10.1002/nap2.70349

**Published:** 2026-11-01

**Authors:** Binbin Zhang, Qingyuan Hu, Xin Liu, Yongyong Zhuang, Yuebin Zhang, Wentao Yang, Wenxu Huang, Zhuo Xu, Xiaoyong Wei

**Affiliations:** ^1^ Electronic Materials Research Laboratory Key Laboratory of the Ministry of Education & International Center for Dielectric Research School of Electronic Information Xi'an Jiaotong University Xi'an China

## Abstract

Owing to their outstanding electro‐optic (EO) coefficients, relaxor ferroelectric crystals are promising candidates for next‐generation integrated optoelectronic devices, particularly high‐performance EO modulators. However, the realization of high‐performance EO modulators relies on high‐quality optical waveguides, which remains a critical challenge for relaxor ferroelectric crystals. Here, we report the fabrication of a planar waveguide in surface layer of the Pb(In_1/2_Nb_2/3_)O_3_–Pb(Mg_1/2_Nb_2/3_)O_3_–PbTiO_3_ (PIN‐PMN‐PT) relaxor ferroelectric single crystal using the He^+^ ion implantation technique. He^+^ implantation generates a subsurface damaged layer confined hundreds of nanometers below the crystal surface within the submicron range, whereas the overlying near‐surface region retains a pristine single‐crystal lattice. Moreover, the energy range of He ion implantation is 100–380 keV, and the damaged layer exhibits a reduced refractive index (maximum Δ*n* ≈ 2.8%), which supports well‐defined waveguide modes in the surface crystalline layer, as proved in the prism coupling measurement. The reconstructed refractive index profiles of the He^+^‐implanted waveguide exhibit a characteristic optical barrier structure. This work not only clarifies the effects of He^+^ ion implantation on the structural properties of the PIN‐PMN‐PT single crystal but also provides an effective strategy for fabricating surface planar optical waveguides, thereby addressing a critical bottleneck for the application in integrated EO modulators.

## Graphical abstract

Helium‐ion implantation turns bulk PIN‐PMN‐PT relaxor ferroelectric crystals into high‐quality planar optical waveguides. The guiding surface layer retains its single crystallinity and large electro‐optic response without postimplantation annealing, whereas a buried damage layer acts as the optical barrier. The waveguides support both single‐ and few‐mode guidance at 633 nm and stable single‐mode guidance at 980 and 1550 nm, opening a direct route to integrated electro‐optic modulators.

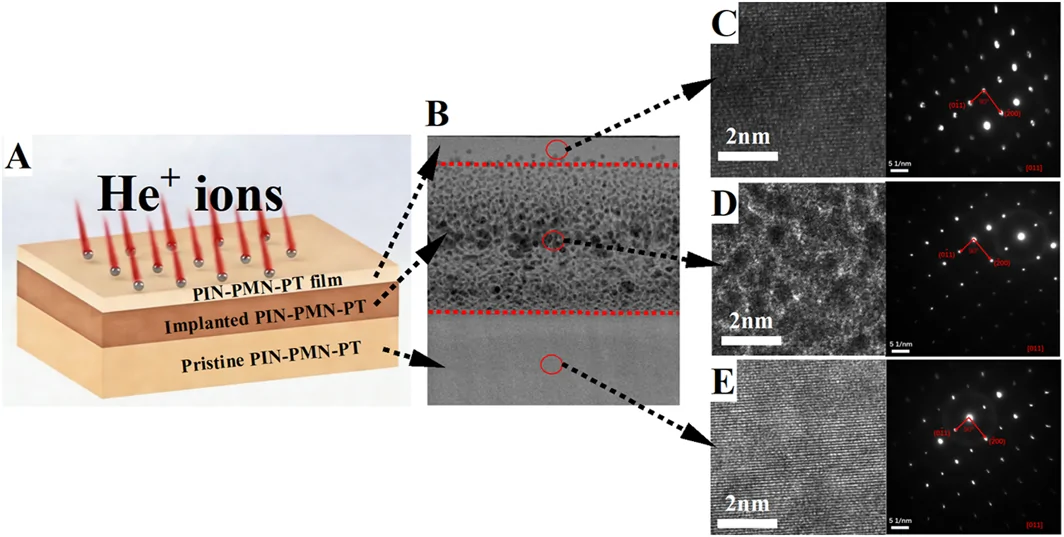

## 1 Introduction

Lead‐based relaxor ferroelectric single crystals, including the Pb(Mg_1/2_Nb_2/3_)O_3_–PbTiO_3_ (PMN‐PT) and Pb(In_1/2_Nb_2/3_)O_3_–Pb(Mg_1/2_Nb_2/3_)O_3_–PbTiO_3_ (PIN‐PMN‐PT) single crystal, have attracted extensive attention owing to their exceptional piezoelectric and electromechanical coupling coefficients (*d*_33_ > 1500 pC/N, *k*_33_ > 90%) [1, 2, 3, 4]. These superior properties have enabled their widespread applications in medical ultrasonic diagnostics, underwater acoustics, and precision actuation [5, 6, 7]. Beyond the excellent electromechanical performance, lead‐based relaxor ferroelectric single crystals also exhibit extraordinary electro‐optic (EO) effects, which can exceed 200 pm/V, approximately one order of magnitude higher than those of conventional EO crystals, such as LiNbO_3_ and DKDP, demonstrating great potential for next‐generation photonic applications [8, 9].

Research on the EO properties of relaxor ferroelectrics dates back to the pioneering work of Smolenskii et al. in the 1970s, which focused on thermal stability [10, 11]. Subsequent milestones include the observation of a pronounced photorefractive effect in PMN‐0.38PT single crystals [12, 13] and systematic characterizations of the transparency and refractive index of PIN‐PMN‐PT single crystals [14, 15, 16, 17]. To mitigate domain‐wall‐originated optical scattering losses, Liu et al. developed a “2R” domain engineering approach by high‐temperature poling along the [011] crystallographic direction for PIN‐PMN‐PT single crystals, which eliminates optical scattering and elevates the peak transmittance to 99.6% [4].

Optical waveguides represent the fundamental building blocks of integrated photonics, providing a high‐refractive‐index region required to confine light within subwavelength volumes [18]. Considerable efforts have been devoted to fabricating waveguides in PIN‐PMN‐PT crystals. Hu et al. demonstrated a titanium‐diffused planar waveguide in PIN‐PMN‐PT single crystals, achieving a refractive index change of approximately 3% [19, 20]. However, metal in‐diffusion may theoretically degrade the single‐crystalline structure and thus impair the functional performance of the material. Therefore, the development of alternative fabrication approaches is highly desirable.

Ion implantation has emerged as a powerful technique for constructing waveguide structures, which enables precise, low‐temperature control over the depth and concentration of implanted ions [21, 22, 23, 24, 25]. Implantation of light ions (e.g., He^+^) typically generates a buried low‐refractive‐index “optical barrier” via nuclear damage at the end of the ion trajectory. To suppress evanescent field tunneling into the substrate, multi‐energy implantation is often adopted to broaden the barrier layer [26, 27]. To date, ion implantation has been successfully applied to more than 100 material systems, including single crystals, ceramics, and glasses [28]. Moran et al. reported a refractive index change (Δ*n*) of nearly 18% in PZN‐PT crystals induced by He^+^ ion implantation, demonstrating the feasibility of waveguide formation [29]. Nevertheless, detailed structural analysis on the influence of ion implantation is not given in their work, which provides limited guidance for constructing functional optical waveguide structures. An actual PIN‐PMN‐PT ridge‐waveguide electro‐optic modulator has already been demonstrated via a top‐down etching and thin‐film process [30]. By contrast, the present work focuses on a buried planar waveguide formed directly in bulk PIN‐PMN‐PT by He^+^ implantation, a complementary platform that also serves as the foundation for subsequent ridge‐waveguide and modulator fabrication. H^+^ implantation has recently been explored as a smart‐cut route to nanoscale single‐crystal films [31], since postimplantation annealing generates dense H_2_ bubbles that drive layer splitting. He^+^ implantation, however, does not form such bubbles upon annealing and thus cannot be used for layer transfer; instead it leaves a permanent, radiation‐damaged near‐surface layer that acts as the optical barrier underlying the planar waveguides reported here.

Here in this work, surface planar optical waveguides are successfully fabricated in PIN‐PMN‐PT single crystals via He^+^ ion implantation. The correlation between implantation energy and ion penetration depth is systematically investigated. By combining dark‐mode spectroscopy (prism coupling) with the reflectivity calculation method (RCM), the extraordinary refractive index profiles are reconstructed and the mode confinement characteristics under different implantation energies are evaluated. This study establishes a feasible route for developing high‐quality waveguides in relaxor‐PT single crystals.

## 2 Results and Discussion

### 2.1 Characterization on the PIN‐PMN‐PT Single Crystal

High optical transmittance and strong electro‐optic (EO) response are essential prerequisites for the PIN‐PMN‐PT single crystal applied in integrated electro‐optic modulators. Thus, the intrinsic optical and electro‐optic (EO) properties of poled PIN‐PMN‐PT crystals were systematically characterized. As shown in Figure 1A, the PIN‐PMN‐PT crystals were cut into rectangular samples with the *x*, *y*, and *z* axes along the [100], [01–1], and [011] directions, respectively. Samples were poled along [0111], with light propagation along the [100] direction. After poling, the transmittance along the [100] (light propagation) and [01–1] (polarization) orientations reached approximately 70%, approaching the theoretical transmittance limit indicated by the dashed line in Figure 1B. Moreover, the transmittance spectrum is nearly flat across the measured wavelength range, indicating negligible absorption, especially at the commonly used wavelengths such as 1064 and 1310 nm [32].

**FIGURE 1:**
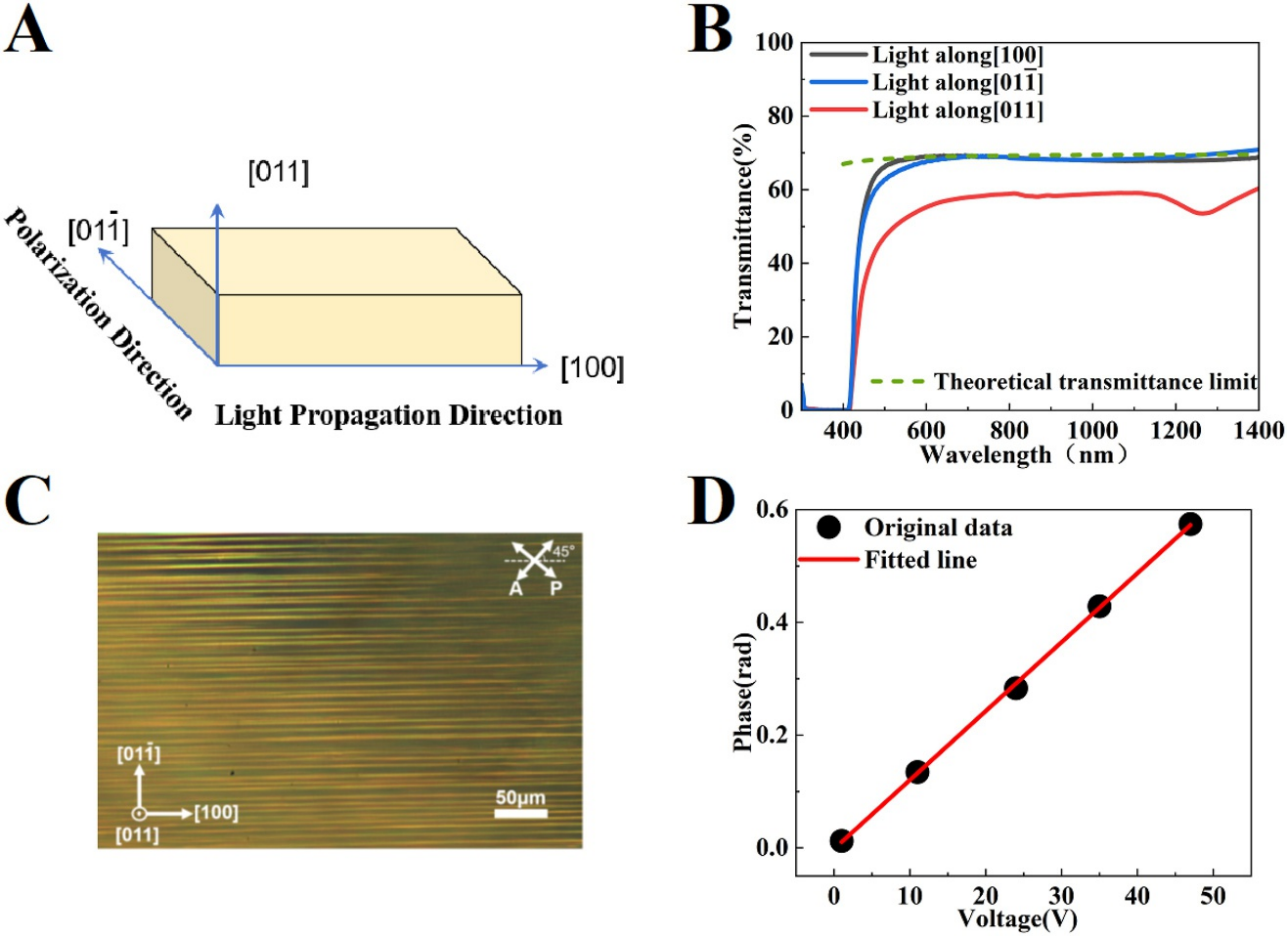
(A) Poling ([01–1]) and light propagation ([100]) directions of PIN‐PMN‐PT crystals. (B) Optical transmittance spectrum of poled PIN‐PMN‐PT. (C) Orthogonal polarized microscopy image of the (011) plane. (D) Phase–voltage fitting curve for EO coefficient measurement.

For relaxor ferroelectric single crystals, the dominant factor limiting transmittance is strong optical scattering at specific domain walls [33]. Generally, transmittance close to the theoretical limit requires a single‐domain state with all domain walls eliminated. However, the single‐domain PIN‐PMN‐PT single crystal typically exhibits relatively low EO coefficients. On the other hand, well‐engineered multi‐domain structures can also achieve near‐ideal transmittance [4]. The strong light scattering at specific domain walls arises from refractive index differences across these boundaries [34]. For the PIN‐PMN‐PT single crystal, a “2R” (twofold rhombohedral) engineered domain structure is formed after high‐temperature poling treatment, containing only two types of ferroelectric domains oriented along [111] and [−111], which form 71° domain walls along the (011) crystallographic plane, as shown in Figure 1C. As the refractive indices on both sides of a domain wall are matched, the optical scattering can be greatly suppressed.

The EO coefficient was measured using a single‐beam polarizer–sample–compensator–analyzer (PSCA) system, or the so‐called Senarmont compensator method [19]. The effective electro‐optic coefficient *γ*_c_ was calculated using the formula [28].

(1) $$\gamma_{c}=\frac{2A\beta_{E}}{V_{dc}}$$

Here, *β*_E_ represents the change in the azimuth angle of the polarizer, *V*_dc_ is the applied electric field, and *A* is a constant dependent solely on the parameters of the crystal, with a value of:

(2) $$A=\frac{d\lambda }{L{\pi n}^{3}}$$

Based on the linear fitting shown in Figure 1D, the measured value of *β*_E_ was determined to be 0.01223. Using Formula (2), it was found that the effective electro‐optic coefficient *γ*_c_ is 509 pm/V. This value is 25.5 times larger than that of conventional LiNbO_3_ single crystals (approximately 20 pm/V) [35]. The high optical transmittance and excellent EO coefficients of the poled PIN‐PMN‐PT crystals indicate a significant potential for application in waveguide modulators.

### 2.2 Design and Simulation of He^+^‐Implanted Waveguides

As mentioned above, the fabrication of a high‐quality optical waveguide is critical for the application of PIN‐PMN‐PT single crystals. Herein, the He^+^ ion implantation was selected as a low‐damage, precise fabrication strategy. To further elucidate the He^+^ ion implantation process in PIN‐PMN‐PT single crystals, Stopping and Range of Ions in Matter (SRIM) simulations were performed to predict the ion penetration, damage distribution, and refractive index modulation, which provides a theoretical guideline for experimental parameters.

Figure 2 systematically characterizes the simulated ion range, energy loss, ion distribution, and lattice damage evolution at implantation energies of 100, 200, 300, and 380 keV. As shown in Figure 2A, the projected ion range (implantation depth) in the PIN‐PMN‐PT single crystals increases nearly linearly with implantation energy, advancing from ∼0.38 μm at 100 keV to ∼0.94 μm at 380 keV. This trend aligns with fundamental ion–solid interaction theory and enables precise control over the waveguide depth—a critical requirement for constructing planar waveguides with tailored optical confinement.

**FIGURE 2:**
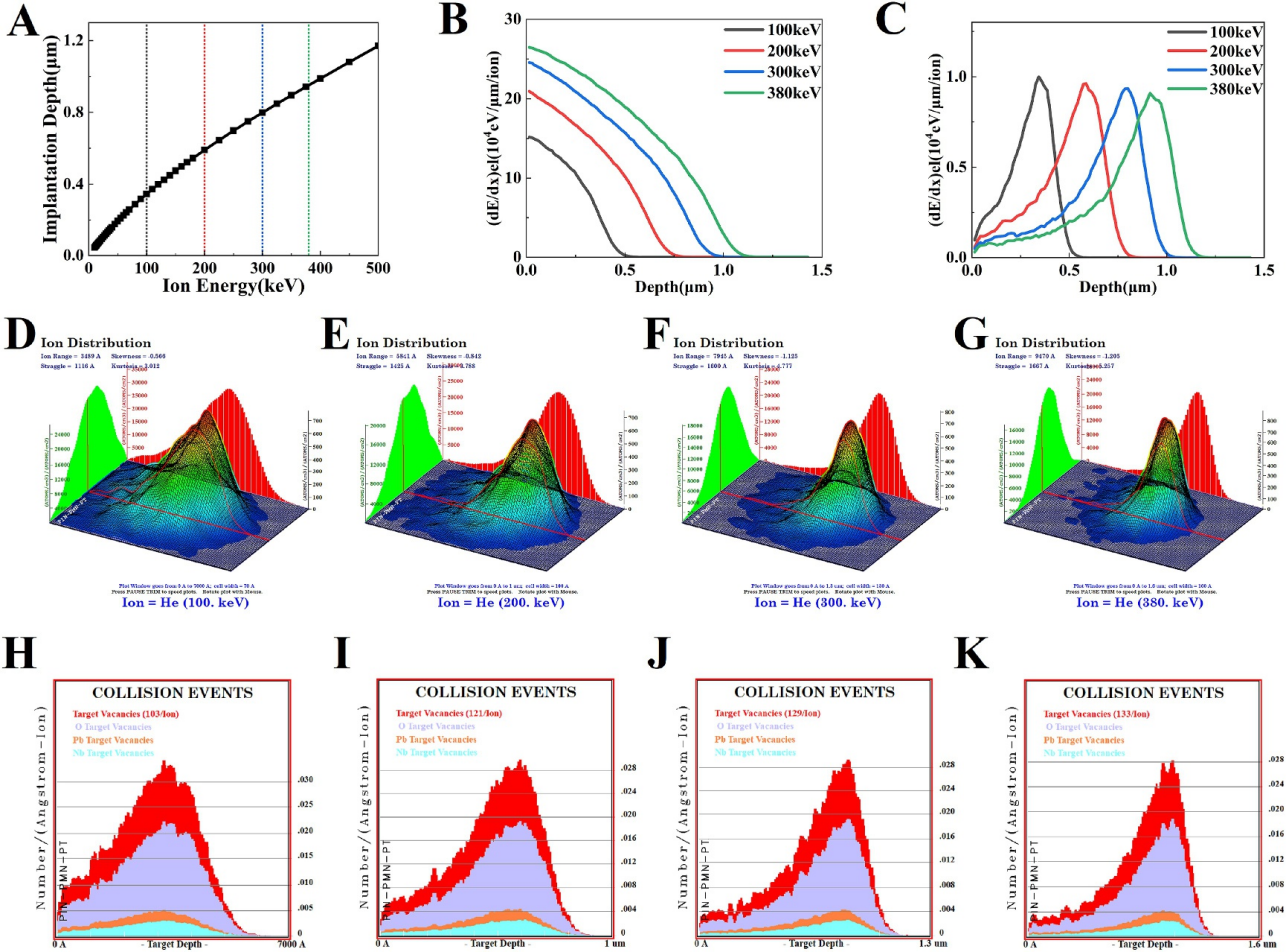
(A) Implantation depth versus He^+^ energy. (B,C) Electronic and nuclear energy loss profiles at different energies. (D–G) He^+^ ion spatial distributions (100–380 keV). (H–K) SRIM‐simulated target vacancy distributions (100–380 keV).

The energy loss of incident He^+^ ions occurs via two distinct mechanisms: electronic and nuclear energy loss (Figure 2B,C, respectively). Electronic energy loss stems from inelastic collisions with target electrons, causing electron excitation and ionization. Conversely, nuclear energy loss originates from elastic collisions with target nuclei, which directly displace lattice atoms and generate irradiation‐induced defects [36]. As depicted in Figure 2C, the nuclear energy loss exhibits a distinct Gaussian peak near the end of the ion range, directly corresponding to the region of maximum lattice damage.

Figure 2D–G illustrate the three‐dimensional spatial distributions of the He^+^ ions at the various implantation energies. The ion concentration profiles exhibit a typical Gaussian distribution, with the concentration peak shifting deeper as the implantation energy increases. Concurrently, the ion straggle (distribution width) broadens at higher energies due to enhanced multiple scattering during lattice transport.

Figure 2H–K display the SRIM‐simulated target vacancy distributions (collision events) induced by He^+^ implantation. The total vacancy concentration (red area) exhibits a distinct damage peak near the end of the ion range, completely consistent with the nuclear energy loss profile shown in Figure 2C. The individual O, Pb, and Nb vacancies (purple, orange, and cyan, respectively) follow this identical depth‐dependent trend, with the peak damage position shifting monotonically deeper at higher implantation energies. Crucially, this damage architecture provides the physical mechanism for refractive index modification in ion‐implanted waveguides: the buried damage peak creates a low‐refractive‐index optical barrier, whereas the minimally damaged near‐surface layer retains a high refractive index, thereby effectively confining light propagation within the waveguide core.

The simulations indicate that high‐energy ion bombardment induces two successive energy transfer stages, yielding a characteristic damage profile within the surface layer of the PIN‐PMN‐PT single crystal. In the electronic stopping regime, high‐velocity incident ions penetrate the material and primarily undergo inelastic collisions with target electrons [37]. This energy transfer causes electron excitation and ionization but produces minimal atomic displacement. Consequently, the near‐surface layer sustains negligible structural damage, leaving its intrinsic refractive index largely unperturbed.

As the ion velocity decreases, the system enters the nuclear stopping regime, where elastic collisions with target nuclei dominate. Momentum is directly transferred to the lattice atoms; when the transferred energy exceeds the displacement threshold, atoms are ejected from their equilibrium sites, creating Frenkel defects (interstitial–vacancy pairs). Upon coming to rest, the implanted ions form a buried doped region, whereas the heavily accumulated defects generate a pronounced damage peak near the end of the ion range. This buried damage peak functions effectively as an optical cladding layer, facilitating the formation of a planar optical waveguide within the well‐preserved near‐surface region of the crystal.

Guided by these SRIM simulations, He^+^ implantation experiments were conducted. The cross‐sectional microstructure of the implanted crystals was subsequently characterized using transmission and scanning electron microscopy (TEM/SEM) to experimentally verify the predicted layered architecture and damage depth.

### 2.3 Microstructure of He^+^‐Implanted PIN‐PMN‐PT

A schematic of the He^+^ implantation process is shown in Figure 3A. After implantation, a sandwich‐like layered structure is formed in the crystal, consisting of a near‐surface layer, an intermediate ion‐implanted layer, and the underlying bulk pristine crystal. The microstructure of this layered structure was characterized by high‐resolution TEM, as presented in Figure 3B. A nano‐porous structure is clearly observed in the region beneath the crystal surface. During implantation, incident ions collide with lattice atoms, producing point defects and lattice distortion at a characteristic depth (i.e., the ion range). The formation of this porous‐like structure can thus be attributed to structural damage caused by the stopping and energy deposition of implanted ions.

**FIGURE 3:**
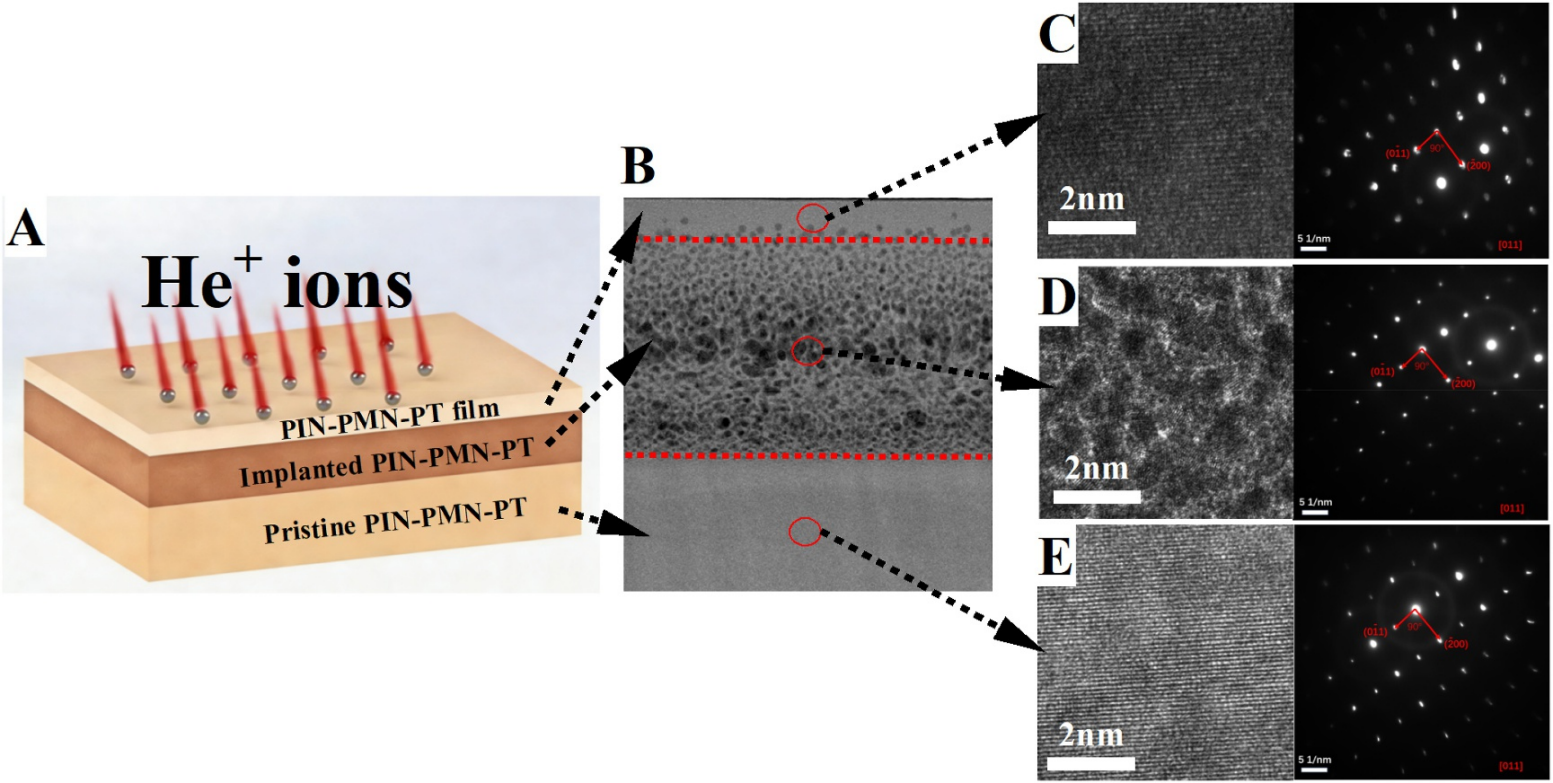
(A) Schematic of He^+^ implantation for waveguide fabrication. (B) Cross‐sectional TEM of He^+^‐implanted PIN‐PMN‐PT. (C–E) High‐magnification TEM and SAED of surface, implanted, and pristine layers.

TEM images of representative regions of different layers are shown in Figure 3C–E. Both the surface layer and the original bottom layer show highly ordered lattices and superior crystallinity, whereas the crystal structure is severely damaged in the ion‐implanted middle layer. Specifically, clear, sharp, and regularly arranged electron diffraction spots are observed in the selected‐area electron diffraction (SAED) patterns of all three regions (surface layer, implanted layer, and pristine layer), indicating a well‐ordered crystalline structure. The absence of visible defects in the SAED pattern of the implanted layer is mainly attributed to the small selected area size during SAED characterization, which is insufficient to cover the microscale defects (e.g., lattice distortions, point defects, and dislocations) induced by He^+^ implantation. These defects are typically discretely distributed within the implanted layer, and the limited observation area fails to capture their characteristic diffraction signals, resulting in the absence of defect‐related diffraction anomalies in the SAED patterns.

To investigate the influence of implantation energy on the crystalline structure, He^+^ ion implantation was performed on PIN‐PMN‐PT single crystals at energies of 100, 200, 300, and 380 keV. Cross‐sectional images of the implanted crystals are shown in Figure 4A–D. The cross‐sectional structures of samples implanted at 200, 300, and 380 keV were characterized by SEM. Owing to the relatively thin surface layer of the 100 keV‐implanted sample, its cross‐sectional structure was characterized by TEM. The implantation depth is defined as the distance from the sample surface to the center of the implanted region. As observed from Figure 4A–D, the corresponding implantation depths are 352, 604, 824, and 1042 nm for implantation energies of 100, 200, 300, and 380 keV, respectively. It agrees well with the simulation result in Figure 2A.

**FIGURE 4:**
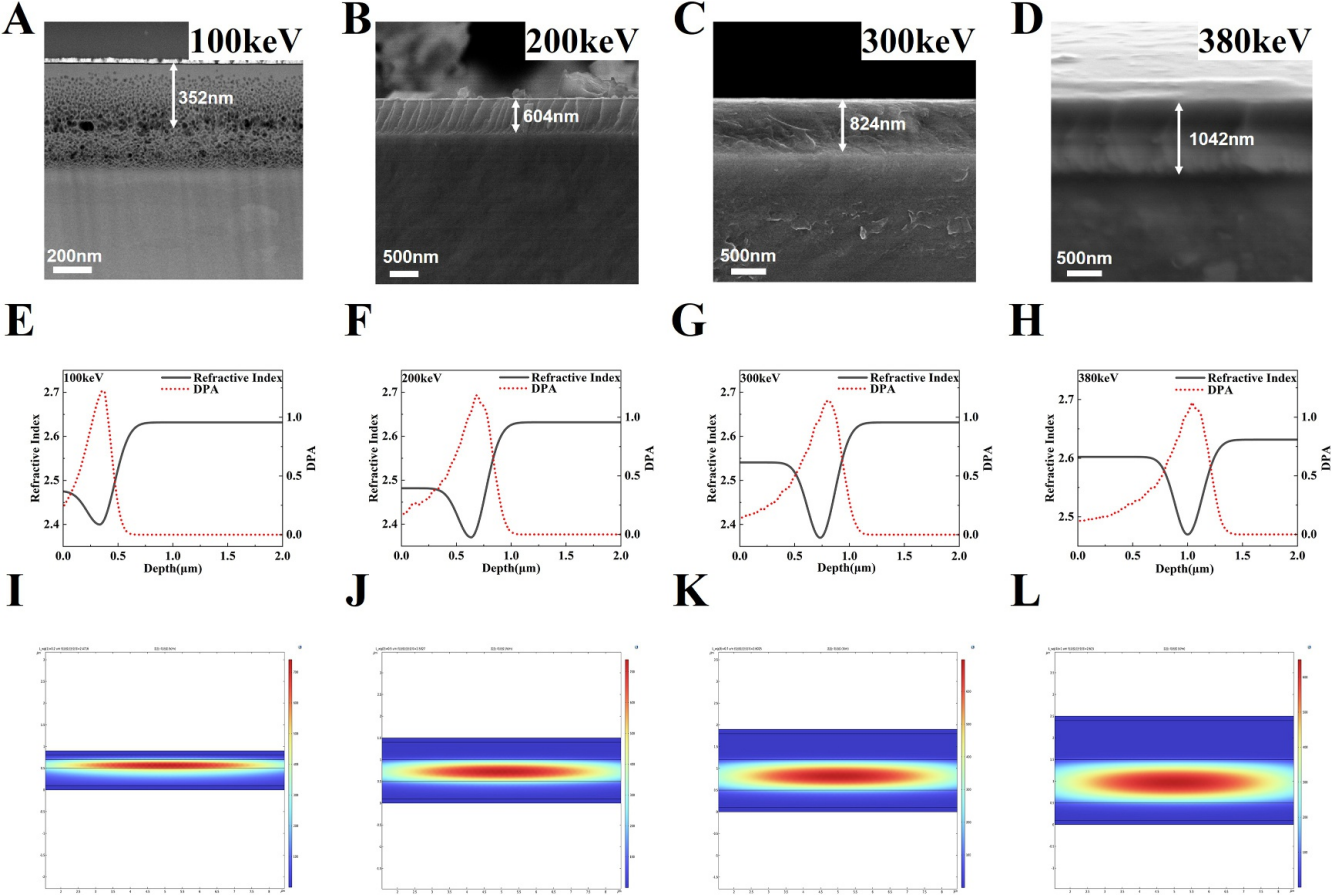
(A–D) Cross‐sectional TEM/SEM of 100–380 keV He^+^‐implanted PIN‐PMN‐PT. (E–H) Refractive index profiles (RCM) and DPA distributions (SRIM). (I–L) FEM‐simulated near‐field intensity of fundamental guided modes.

The buried nanoporous damage layer induced by He^+^ implantation is expected to reduce the refractive index via lattice disorder and defect accumulation. To confirm this and evaluate optical confinement, refractive index profiles were reconstructed by RCM [38], and mode fields were simulated by finite element method (FEM).

The refractive index profile was reconstructed via the RCM, a nondestructive technique that approximates the ion‐implanted waveguide as a multilayer dielectric stack. By calculating Fresnel reflection coefficients and interference effects at each interface, a theoretical reflectance spectrum is generated from an initial index model. Through iterative fitting to the experimental spectrum, model parameters are continuously adjusted until convergence. The final inverted profile accurately describes the depth‐dependent refractive index distribution, facilitating the evaluation of optical confinement and transport properties.

The refractive index profile is a fundamental and critical parameter for characterizing the guiding performance of integrated optical waveguides, yet it has not been systematically investigated for He^+^‐implanted PIN‐PMN‐PT single‐crystal waveguides. In this work, the RCM was employed to reconstruct the extraordinary refractive index (*n*_e_) profiles of the implanted waveguides based on dark‐mode spectra measured by the prism coupler. Figure 4E–H present the reconstructed refractive index distributions (solid black lines) for waveguides fabricated at 100, 200, 300, and 380 keV, with the red dashed lines representing SRIM‐simulated displacement per atom (DPA) profiles (a quantitative measure of nuclear damage) for comparison.

The deviation between experimentally measured and theoretically calculated effective refractive indices of dark modes is within 10^−4^ for all samples, confirming the high reliability and accuracy of the RCM reconstruction. As shown in the figures, all implanted samples exhibit a characteristic buried optical barrier refractive index profile, which is a typical structural feature of ion‐implanted dielectric waveguides. The near‐surface region retains a high refractive index close to the bulk value, acting as the waveguide core for light confinement, whereas a distinct low‐refractive‐index layer forms at a certain depth beneath the surface, serving as the optical cladding to prevent light leakage into the substrate.

A clear monotonic correlation between implantation energy and barrier characteristics is observed across all samples: the depth of the low‐index barrier increases progressively with rising implantation energy, whereas the magnitude of the refractive index reduction first increases and then slightly decreases at the highest energy. The SRIM‐simulated DPA profiles, which quantify lattice displacement induced by ion implantation, show distinct Gaussian‐shaped nuclear damage peaks at depths that align precisely with the low‐index barrier positions reconstructed by RCM. This strong correlation directly confirms the physical mechanism of refractive index modification in He^+^‐implanted PIN‐PMN‐PT crystals: nuclear energy loss during implantation induces lattice displacement, point defects, and local structural disorder within the ion range, which reduce material density and thus lower the refractive index, forming the buried optical barrier.

These simulation results are also highly consistent with the damage depths measured by TEM and SEM in previous sections, further validating the reliability of the refractive index reconstruction and the damage mechanism. The ability to precisely control the depth and magnitude of the refractive index barrier by adjusting the implantation energy provides a flexible approach for tailoring the mode field distribution and optical confinement of PIN‐PMN‐PT waveguides, which is of great significance for the development of high‐performance integrated EO devices based on this material system.

Figure 4I–L present FEM simulations of the near‐field light intensity distributions for the fundamental guided modes in the He^+^‐implanted PIN‐PMN‐PT planar waveguides, performed using COMSOL Multiphysics software based on the experimentally reconstructed refractive index profiles. The simulation results clearly show that all waveguides exhibit excellent optical confinement performance: light energy is tightly localized within the high‐refractive‐index near‐surface waveguide core, with negligible leakage into the underlying low‐refractive‐index damage layer and bulk substrate. As the implantation energy increases from 100 to 380 keV, the waveguide core thickness gradually increases and the mode field distribution expands correspondingly, which shows that it is directly correlated with the deepening of the buried low‐index optical barrier. This excellent optical confinement is a fundamental prerequisite for the development of high‐performance EO modulators and other integrated photonic devices based on PIN‐PMN‐PT single crystals.

### 2.4 Structural Stability and Ferroelectric Domain Preservation

To elucidate the structural evolution of the PIN‐PMN‐PT single‐crystal surface layer before and after He^+^ ion implantation, a series of structural characterizations were performed, which is critical for verifying the retention of the intrinsic high EO properties of the waveguide core. Figure 5 presents the comprehensive structural analysis results, including grazing‐incidence X‐ray diffraction (GI‐XRD), Raman spectroscopy, and piezoresponse force microscopy (PFM) characterizations.

**FIGURE 5:**
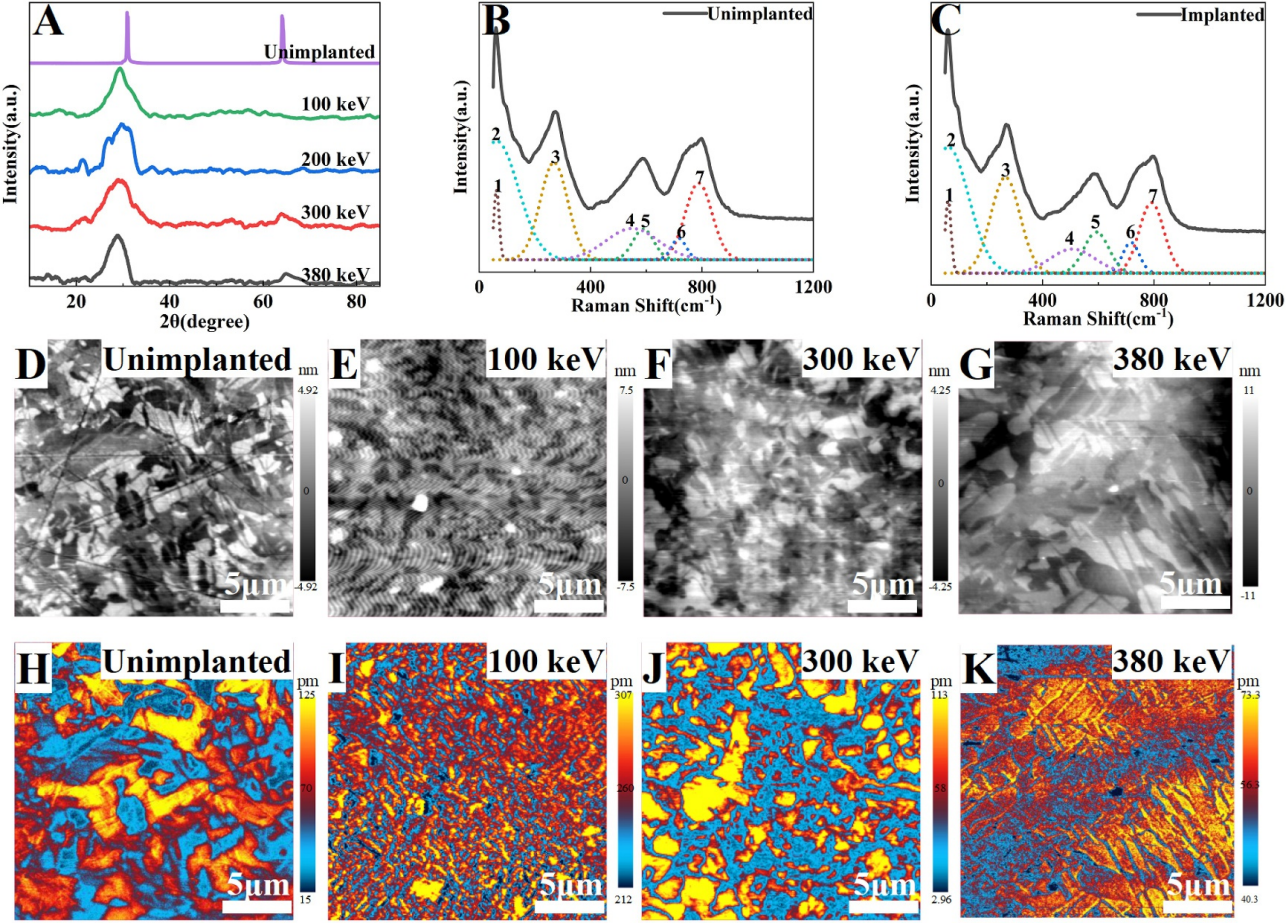
(A) GI‐XRD patterns of pristine and implanted PIN‐PMN‐PT. (B,C) Raman spectra of pristine and implanted crystals. (D–K) PFM topography and amplitude images of pristine and 100/300/380 keV implanted samples.

Figure 5A shows the GI‐XRD patterns of the unimplanted sample and He^+^‐implanted samples at energies ranging from 100 to 380 keV, focusing on the (011) crystallographic plane. The unimplanted sample exhibits a sharp, narrow diffraction peak with a high signal‐to‐noise ratio, confirming excellent long‐range crystalline order and high‐quality single‐crystalline structure. After ion implantation, the diffraction peaks of the 100, 200, and 300 keV samples exhibit obvious broadening (increased FWHM) and reduced intensity, which is a direct signature of lattice distortion, point defect accumulation, and localized structural damage induced by ion irradiation in the near‐surface region. Notably, as the implantation energy increases to 380 keV, the diffraction peak recovers its sharpness and intensity, indicating partial restoration of the crystalline structure in the surface layer. This recovery is attributed to the mild in situ thermal annealing effect induced by high‐energy implantation: under a beam current density of 400 μA/cm^2^, the sample temperature rises only to approximately 70°C, yet this modest heating is sufficient to promote the annihilation of irradiation‐induced defects and the restoration of lattice order, thereby ensuring the retention of the high EO response of the waveguide core.

Figure 5B–C display the Raman spectra of the unimplanted and implanted PIN‐PMN‐PT crystals, respectively. Both spectra exhibit seven characteristic vibrational modes with nearly identical peak positions and relative intensities, consistent with the typical phonon modes of rhombohedral‐phase PIN‐PMN‐PT single crystals. The high consistency of the Raman signals confirms that the detected signal originates primarily from the near‐surface waveguide layer rather than the deeply buried damage zone. This result demonstrates that the crystal structure of the surface waveguide core is well preserved after ion implantation, in excellent agreement with the theoretical expectation that the near‐surface region experiences minimal nuclear damage and retains bulk‐like properties.

Figure 5D–K present the PFM topography and amplitude images of the unimplanted sample and samples implanted at 100, 300, and 380 keV, which directly reflect the evolution of the ferroelectric domain structure. The unimplanted sample shows a typical large‐scale domain morphology characteristic of rhombohedral‐phase PIN‐PMN‐PT single crystals, with clear domain boundaries and uniform domain distribution. After 100 keV ion implantation, the domain size decreases significantly and the domain structure becomes highly fragmented, closely correlated with irradiation‐induced lattice damage and internal stress in the near‐surface layer. As the implantation energy increases to 300 and 380 keV, the domain size gradually recovers and the domain structure returns to a more ordered state, consistent with the structural recovery observed in the GI‐XRD results. This domain evolution is attributed to the dynamic balance between ion irradiation‐induced defect generation and high‐energy implantation‐induced thermal annealing, which modulates the internal stress and domain stability in the surface layer.

All structural characterization results collectively demonstrate that the crystal surface layer of PIN‐PMN‐PT single crystals retains its original single‐crystalline structure and ferroelectric domain characteristics after He^+^ ion implantation, without the need for any additional postimplantation annealing, thereby preserving the intrinsic high EO properties required for high‐performance waveguide devices. This structural stability provides a solid material foundation for the fabrication of integrated EO modulators and other functional devices based on ion‐implanted PIN‐PMN‐PT waveguides.

The present study demonstrates that He^+^ ion implantation can effectively modify the refractive index of PIN‐PMN‐PT single crystals, forming a waveguide region in the near‐surface layer with a higher refractive index than the underlying radiation‐damaged barrier layer. This refractive index modification behavior is consistent with the findings reported by Moran et al. in their investigation of ion‐implanted PZN‐PT single crystals, confirming the universality of this phenomenon in relaxor ferroelectric single‐crystal systems. Beyond this consistency, the present work further elucidates the fundamental mechanism of optical waveguide formation via He^+^ ion implantation and quantitatively characterizes key parameters such as the depth‐dependent refractive index profile of the waveguide, filling a critical knowledge gap in the field of ion–solid interactions for PIN‐PMN‐PT single crystals. These findings are of great significance for the practical application of ion‐implanted PIN‐PMN‐PT optical waveguides. Although favorable optical guiding properties were achieved, preserving the intrinsic single‐crystalline structure and ferroelectric domains is critical for retaining the ultrahigh EO coefficient of PIN‐PMN‐PT.

### 2.5 Optical Waveguiding Performance

The effective modes of the ion‐implanted waveguides were analyzed using a prism coupler. The sample was pressed against a high‐index prism to enable photon tunneling via the evanescent field generated by total internal reflection. At angles satisfying the phase‐matching condition, energy coupling into guided modes produces distinct dips in the angle‐dependent reflection spectrum. Analysis of these coupling resonances yields the effective refractive indices and mode orders, which serve as key indicators of optical confinement and lattice uniformity.

Detailed prism coupling results are presented in Figure 6A–E. For comparison, the unimplanted crystal was also characterized. As shown in Figure 6A, at a wavelength of 633 nm, only one apparent dip is observed in the relative light intensity spectrum, corresponding to a refractive index of 2.63181. The light intensity spectrum of the 100 keV‐implanted crystal is shown in Figure 6B. Two dips are visible, corresponding to refractive indices of 2.47627 and 2.40534, respectively. The shallow slope of these dips indicates severe optical field leakage and the absence of effective waveguide modes. This phenomenon is attributed to the excessively low implantation energy, which results in an overly thin surface waveguide layer unable to effectively confine the optical field.

**FIGURE 6:**
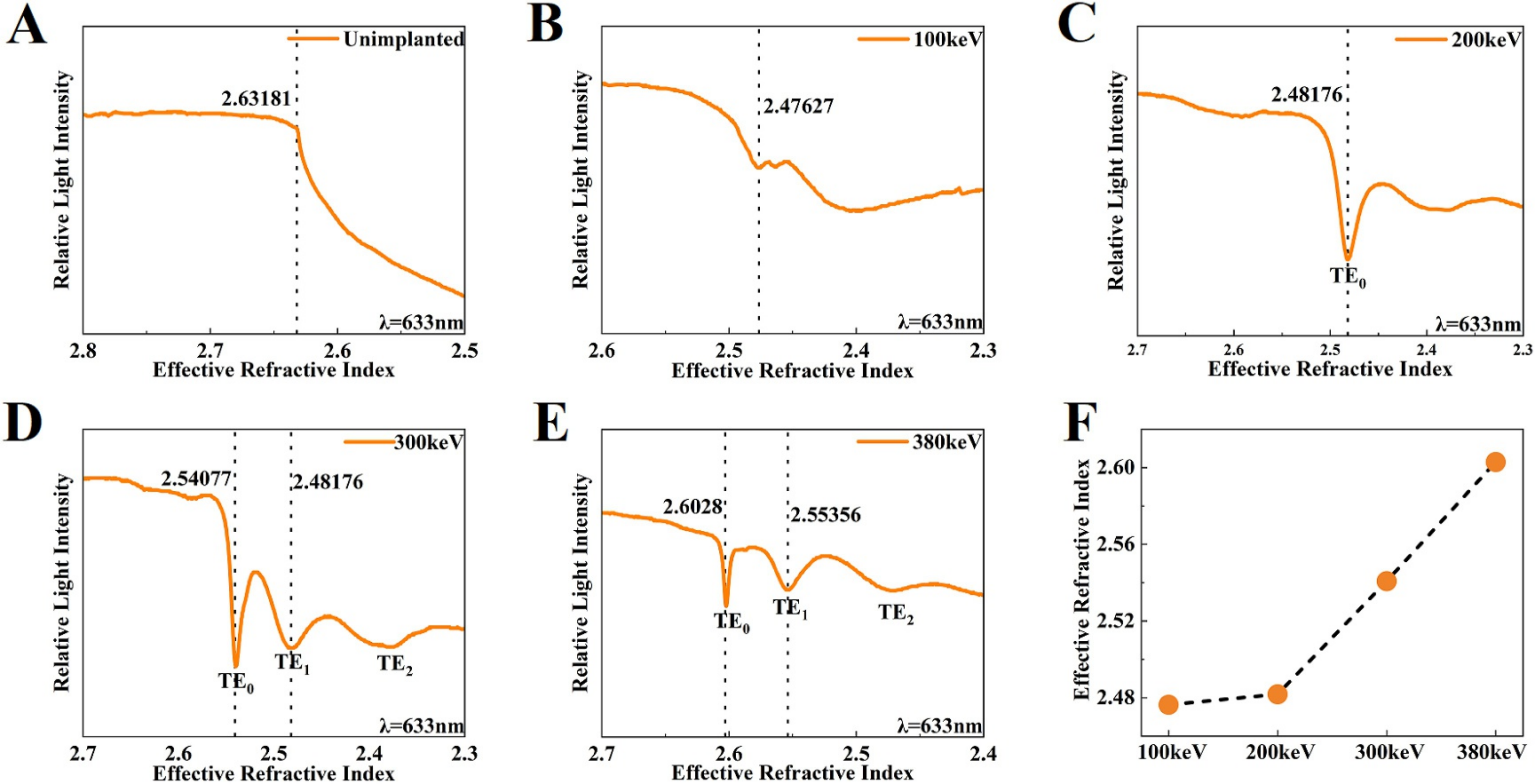
(A–E) Prism coupling spectra (TE polarization) of pristine and 100–380 keV implanted samples at 633 nm. (F) Effective refractive index of fundamental mode versus implantation energy.

Figure 6C shows the light intensity spectrum of the 200 keV‐implanted crystal. A sharp dip is clearly observed, corresponding to a refractive index of 2.48176, which represents the effective refractive index of the fundamental waveguide mode. A broad peak behind the dip corresponds to the coupling signal of high‐order modes. However, the broad peak shape indicates excessive optical field leakage, preventing the formation of effective high‐order modes.

Two sharp dips are observed in the spectrum of the 300 keV‐implanted crystal (Figure 6D), corresponding to the fundamental TE_0_ mode and the first‐order TE_1_ mode, with effective refractive indices of 2.54077 and 2.48176, respectively. Similarly, two sharp dips appear in the spectrum of the 380 keV‐implanted crystal (Figure 6E), corresponding to the TE_0_ fundamental mode and TE_1_ first‐order mode, with effective refractive indices of 2.6028 and 2.55356, respectively.

The relationship between the effective refractive index of waveguide modes and implantation energy is summarized in Figure 6F. The effective refractive index of the fundamental mode increases with increasing implantation energy. Notably, at 380 keV, the effective refractive index of the fundamental mode approaches that of the bulk PIN‐PMN‐PT crystal, indicating excellent optical field confinement with energy tightly localized in the waveguide region and extremely low leakage loss.

The 380 keV upper limit was selected because the resulting core thickness supports 2–3 guided modes, which is sufficient for high‐performance planar waveguides; higher energies would over‐thicken the core, excite unwanted higher‐order modes, and broaden the damage profile, degrading crystallinity and increasing propagation loss. In addition, 380 keV is the standard upper limit of the implanter used, and higher energies fall outside the scope of this study. Consequently, the near‐surface region exhibits a slightly elevated refractive index, whereas the heavily damaged end‐of‐range layer undergoes a significant index reduction. This refractive index profile creates a high‐index guiding core confined between the low‐index optical barrier and the air–crystal interface. This structure supports low‐loss light propagation via total internal reflection while preserving the intrinsic EO properties of the single crystal.

Looking forward, the ion‐implanted PIN‐PMN‐PT surface planar waveguides demonstrated in this work can serve as a foundational platform for the fabrication of advanced functional devices. By integrating complementary micro–nano fabrication technologies such as dry etching, photolithography, and thin‐film deposition, high‐performance ridge waveguides, EO modulators, and integrated photonic chips can be developed. Further details on the electro‐optic modulator design simulations and the prism‐coupling characterization of the implanted waveguides are provided in the Supporting Information S1: Figures S1 and S2. Leveraging the exceptional EO, piezoelectric, and ferroelectric properties of PIN‐PMN‐PT single crystals, these devices hold great potential for applications in high‐speed optical communication, microwave photonics, and integrated quantum photonics.

## 3 Conclusions

In summary, surface planar optical waveguides are successfully fabricated in 25PIN–44PMN–31PT single crystals via He^+^ ion implantation. The effects of implantation energy (100–380 keV) on waveguide performance are systematically investigated. Structural analyses confirm that ion implantation induces localized lattice damage, whereas the near‐surface layer retains good crystallinity. Optical measurements reveal an energy threshold for effective waveguiding: energies ≥ 200 keV support stable guided modes, and 300/380 keV produce well‐defined TE_0_/TE_1_ modes with strong optical confinement. Refractive index profiles show a typical optical barrier structure, consistent with SRIM simulations and TEM/SEM observations. The 380 keV implantation yields optimal performance, with minimal leakage loss and an effective index close to the bulk value. This work demonstrates that He^+^ implantation is an effective strategy for PIN‐PMN‐PT waveguides, providing a solid basis for integrated electro‐optic modulators.

## 4 Experimental Section

The substrate used in this study was a 25Pb(In_1_/_2_Nb_1_/_2_)O_3_‐44Pb(Mg_1_/_3_Nb_2_/_3_)O_3_‐31PbTiO_3_ (25PIN‐44PMN‐31PT) single crystal grown by the Bridgman–Stockbarger method [39]. The crystal dimensions were 20 (*x*) × 16 (*y*) × 1 (*z*) mm^3^, with all surfaces optically polished to ensure efficient light coupling.

Waveguide fabrication began with high‐quality surface preparation: the samples were meticulously polished to obtain a smooth surface, minimizing scattering losses and ensuring efficient light propagation within the waveguide. After polishing, the samples were annealed to eliminate residual stresses and defects introduced during polishing, and to restore the lattice integrity of the crystal, which is critical for reducing optical losses in the waveguide. To determine the optimal annealing conditions, sequential annealing treatments (200°C–800°C, air, 2 h) were performed to optimize surface quality.

Planar optical waveguides were fabricated via He^+^ ion implantation on the largest polished surface (20 × 16 mm^2^) at energies of 100, 200, 300, and 380 keV, with a fluence of 5 × 10^16^ ions/cm^2^, to achieve strong optical confinement at the crystal surface. Previous results indicate that 380 keV implantation yields superior waveguide performance. All implantations were performed with the ion beam incident at 7° off the surface normal to minimize channeling effects. During the implantation process, the sample was mounted on a copper sample stage with thermally conductive silver adhesive, and the sample stage was equipped with a circulating water cooling system. The beam current density was controlled below 400 μA/cm^2^, which would not cause thermal damage to the crystal. For 380 keV He^+^ implantation under the above beam conditions, the actual temperature of the sample is less than 70°C, which has no impact on the crystal structure and waveguide performance.

The transmittance of poled crystals was measured using a UV–Vis spectrophotometer, and domain structures were observed under a polarizing microscope. The EO coefficients of poled crystals were determined using a home‐built EO coefficient measurement system. Cross‐sectional samples for scanning electron microscopy (SEM) and transmission electron microscopy (TEM) were prepared via focused ion beam (FIB) milling, to characterize the microstructural evolution and implantation depth distribution under different implantation energies.

Waveguide properties were investigated using the prism coupling technique. For simplicity and consistency, only transverse electric (TE) modes were analyzed in this work. A Model 2010 Prism Coupler (Metricon) was used to measure the dark‐mode spectra at a wavelength of 633 nm.

In addition, grazing‐incidence x‐ray diffraction (GI‐XRD), Raman spectroscopy, and piezoresponse force microscopy (PFM) were employed to characterize the crystalline and ferroelectric domain structures before and after ion implantation.

To gain in‐depth insight into ion implantation behavior and waveguide performance, numerical simulations and theoretical calculations were performed. The Stopping and Range of Ions in Matter (SRIM) 2013 code was used to simulate the He^+^ ion distribution, damage layer depth, and defect distribution induced by ion implantation, using the full damage cascade mode to ensure simulation accuracy. This method is widely used in ion implantation research to predict ion ranges and radiation damage distributions in target materials, consistent with standard practices in similar ferroelectric crystal studies [40].

The reflectivity calculation method (RCM) was applied to reconstruct the refractive index profile of the implanted planar waveguide, a reliable and widely used technique for determining index distributions in ion‐implanted waveguides [41]. To reveal the intrinsic correlation between radiation damage and refractive index modification, the reconstructed index profiles were systematically compared with SRIM‐simulated damage distributions. This comparison is critical for understanding the refractive index modulation mechanism in PIN‐PMN‐PT crystals, since ion‐induced defects and lattice damage dominate the index contrast between the waveguide core and cladding.

Furthermore, COMSOL Multiphysics software was used to simulate the effective refractive indices of guided modes and the near‐field optical intensity distributions of the planar waveguides based on the finite element method (FEM). The FEM is well recognized for its high accuracy in solving complex electromagnetic field problems in waveguide structures [42]. The simulation adopted a standard dielectric slab waveguide model with reasonable boundary conditions and calculation parameters, enabling reliable prediction of the optical propagation characteristics of the fabricated waveguides.

## Author Contributions

**Binbin Zhang:** writing – original draft. **Qingyuan Hu:** methodology, writing – review and editing. **Xin Liu:** software. **Yongyong Zhuang:** investigation. **Yuebin Zhang:** validation. **Wentao Yang:** Validation. **Wenxu Huang:** validation. **Zhuo Xu:** project administration. **Xiaoyong Wei:** formal analysis.

## Conflicts of Interest

The authors declare no conflicts of interest.

## Supporting information


Supporting Information S1

## Data Availability

The data that support the findings of this study are available from the corresponding author upon reasonable request.
