# Supplementary material for: PIN‐PMN‐PT Surface Planar Waveguides Realized via He+ Implantation
Source: Nanophotonics. 2026 Nov 1;15(21):e70349. doi: 10.1002/nap2.70349 (PMC13724080; doi:10.1002/nap2.70349)
Supplement: Supplementary file 1 [file NAP2-15-e70349-s001.docx]

**Supplementary Material**

**Fig. S1.** (A) Simulated optical mode-field distribution in the ridge-waveguide cross-section. (B) Simulated electric-field distribution (colormap with overlaid field lines) under an applied voltage between the two side electrodes. (C) Contour map of the half-wave voltage–length product Vπ·L as functions of electrode gap and ridge-etch depth.

The optical mode profile of the ridge cross-section was first calculated by a finite-element mode solver (Fig. S1A), showing that the guided field is tightly confined within the ridge core with negligible leakage toward the sidewalls. The corresponding electrostatic simulation under a voltage applied across the two side electrodes is shown in Fig. S1B, where the field lines are predominantly oriented along the electro-optic tensor direction in the waveguide core. Fig. S1C summarizes the resulting half-wave voltage–length product Vπ·L over the design space: smaller electrode gaps and shallower ridge etch depths reduce Vπ·L by improving the overlap between the optical mode and the modulating electric field, whereas wider gaps and deeper ridges progressively degrade the overlap efficiency.

**Fig. S2.** (A–C) Prism coupling spectra (TE polarization) of the 200, 300, and 380 keV implanted samples, respectively, measured at 980 nm. (D–F) The corresponding spectra at 1550 nm. The dips labeled TE₀ denote the fundamental guided mode.

To examine the waveguiding behavior at longer wavelengths, prism-coupling measurements were additionally performed on the same 200–380 keV implanted samples at 980 nm and 1550 nm (Fig. S2). At 980 nm, a well-defined TE₀ resonance is resolved for all three implantation energies, with the effective index increasing monotonically from 2.292 to 2.387 to 2.436 as the energy rises from 200 to 380 keV. For the 380 keV sample, a weaker secondary dip is additionally observed near n_eff_ ≈ 2.33, which can be attributed to a higher-order guided mode. At 1550 nm, clear TE₀ dips are resolved for the 200 and 300 keV samples, yielding effective indices of 2.168 and 2.302, respectively; for the 380 keV sample, the fundamental resonance is markedly weaker and appears only as a shallow shoulder near n_eff_ ≈ 2.361, indicating that the confinement is close to the cutoff. The absence of higher-order modes at 1550 nm is consistent with the shallow modified layer produced by keV-energy ion implantation: the longer wavelength extends the mode field more deeply into the substrate, so that only the strongest (fundamental) confinement remains sufficient to guide light, confirming the single-mode operation of the present waveguides in the near-infrared communication band.
